# Do I Have My Attention? Speed of Processing Advantages for the Self-Face Are Not Driven by Automatic Attention Capture

**DOI:** 10.1371/journal.pone.0110792

**Published:** 2014-10-22

**Authors:** Helen Keyes, Aleksandra Dlugokencka

**Affiliations:** Department of Psychology, Anglia Ruskin University, Cambridge, United Kingdom; Vanderbilt University, United States of America

## Abstract

We respond more quickly to our own face than to other faces, but there is debate over whether this is connected to attention-grabbing properties of the self-face. In two experiments, we investigate whether the self-face selectively captures attention, and the attentional conditions under which this might occur. In both experiments, we examined whether different types of face (self, friend, stranger) provide differential levels of distraction when processing self, friend and stranger names. In Experiment 1, an image of a distractor face appeared centrally – inside the focus of attention – behind a target name, with the faces either upright or inverted. In Experiment 2, distractor faces appeared peripherally – outside the focus of attention – in the left or right visual field, or bilaterally. In both experiments, self-name recognition was faster than other name recognition, suggesting a self-referential processing advantage. The presence of the self-face did not cause more distraction in the naming task compared to other types of face, either when presented inside (Experiment 1) or outside (Experiment 2) the focus of attention. Distractor faces had different effects across the two experiments: when presented inside the focus of attention (Experiment 1), self and friend images facilitated self and friend naming, respectively. This was not true for stranger stimuli, suggesting that faces must be robustly represented to facilitate name recognition. When presented outside the focus of attention (Experiment 2), no facilitation occurred. Instead, we report an interesting distraction effect caused by friend faces when processing strangers’ names. We interpret this as a “social importance” effect, whereby we may be tuned to pick out and pay attention to familiar friend faces in a crowd. We conclude that any speed of processing advantages observed in the self-face processing literature are not driven by automatic attention capture.

## Introduction

We respond more quickly to our own face than to others [1]–[3], but we are unsure of the mechanism underlying this advantage. One sensible suggestion is that the self-face automatically captures our attention, speeding our reaction to it. However, research investigating whether our own face does selectively grab our attention has produced mixed findings, and studies have suffered from a lack of rigorous control in focus of attention. Here, we report two experiments investigating whether the self-face does indeed selectively capture our attention, and the attentional conditions under which this might be possible.

One of the key questions in attention research revolves around how much information we process from stimuli that we are not directly attending to, and the circumstances in which these unattended stimuli can capture our attention (cf. the Cocktail Party phenomenon; [4], [5]). One common way to investigate this question is to present task-irrelevant stimuli and measure their effect on task performance (cf. The Stroop effect; [6]). We can further investigate the phenomenon by measuring the effect of presenting task-irrelevant stimuli both inside and outside the focus of attention, enabling us to define the circumstances in which different classes of task-irrelevant stimuli selectively capture our attention.

### Self-name and attention capture

The idea that self-referential stimuli can selectively capture our attention has largely been studied using the self-name. Several studies show that our own name is processed preferentially, but only when it is task-relevant; our own name does not selectively capture attention when it is irrelevant to the task at hand [7], [8]. Conversely, others show that the self-name does have selective attention-capture capacity, even when task-irrelevant: it is more resistant to the attentional blink, inattentional blindness and repetition blindness than other names and words [9]–[11]. Key to understanding the circumstances in which our own name does automatically capture our attention are studies manipulating the focus of attention.

In an important paper, Gronau and colleagues look at the effects that personally relevant stimuli – self-names – have when presented both inside and outside the focus of attention [12]. Participants were instructed to report the colour of a piece of text, but to ignore the distractor names represented by the text, presented centrally (at the focus of attention; Stroop-like task) or to report the colour of a box presented centrally with distractor names presented peripherally (outside the focus of attention). When presented centrally, the self-name caused significantly more interference on the colour-naming task than other names. However, this effect disappeared when the names were presented peripherally above or below a coloured box, outside the focus of attention. Interestingly, skin conductance responses – taken as a sign of processing a stimulus of personal significance [13] – remained larger for self-names compared to other names for both central and peripheral presentation of the names, suggesting that the peripherally presented stimuli were being processed. It seems that the capacity of our own name to automatically capture our attention exists in a complicated relationship with the focus of attention.

### Focus of attention – self-face

Self-referential stimuli may capture attention automatically, but to investigate whether this is due to a truly self-referential effect, Devue and Brédart [14] recommend using self-face stimuli rather than self-name stimuli, as the self-face is unique to each person. Indeed, early evidence suggested that the self-face is more easily detected than recently learned faces [1], but that study suffered from the absence of a familiarity control. There are many reasons to believe that our own face is processed as “special”, with evidence of speeded processing [1]–[3], a widely distributed underlying neural network [15]–[20] and a stronger feature-based processing approach relative to other faces [3], [21]–[23]. Our own face provides a truly unique stimulus, which appears to receive special treatment in the brain. Considering this special treatment, the self-face is a likely candidate to elicit automatic attention capture. Indeed, there is some evidence of automatic processing of the self-face [24], which in turn might indicate an automatic attention capture mechanism. However, although we may pay particular attention to our own face, it is unclear whether the self-face selectively grabs our attention, or whether it simply holds our attention once attended to (see [25]).

Research on whether our own face automatically captures our attention has produced conflicting results. Brédart and Devue [26] conducted one of the first studies looking at the self-face and attention capture. They showed that our own face causes more distraction than other familiar faces when presented peripherally – ostensibly outside of the focus of attention. The authors presented participants with a pair of vertically aligned word stimuli, presented centrally on a screen. Each word pair comprised a name (either the participant’s own name or the name of a familiar classmate) and a letter string. The participant’s task was to indicate whose name was present. Flanking these word pairs either to the left or the right, a distractor picture appeared which showed the participant’s own face, the face of their classmate or the face of their professor. The authors report that the peripheral presentation of the self-face caused more distraction when identifying a classmate’s name than a classmate’s face caused when identifying the participant’s own name, suggesting that the self-face automatically and selectively captured attention, even when presented outside the focus of attention.

A later paper presented conflicting results. Devue and Brédart [14] showed that the self-face and other highly familiar faces produce a temporary distraction when presented inside the focus of attention (between two target digits), suggesting that these types of face can automatically capture attention. However, these faces did not produce a distraction when presented outside the focus of attention. Importantly, the faces presented peripherally were presented only briefly (200 ms), unlike the faces in the earlier study [26], where the stimuli were displayed until the participant responded. Where the stimuli were presented indefinitely, the participant may have had time to shift attention directly towards the distractor faces, bringing them inside the focus of attention. When this factor was adjusted in the later paper [14], self-faces presented peripherally did not selectively capture attention. Further research showed that highly familiar faces (including the self-face) did not reduce inattentional blindness relative to unfamiliar faces, even when presented inside the focus of attention [27]. There remains debate as to whether our own face does selectively capture our attention [26] or whether the self-face is as easily ignored as other familiar [14] and unfamiliar [27] faces. While various methodologies have been employed to investigate this phenomenon, insufficient focus of attention controls may account for many of the reported discrepancies.

### Current Study

The current paper addresses previous issues concerning focus of attention in two ways. First, we use a face-word paradigm to present distractor faces directly central to the focus of attention (Experiment 1) and secondly we employ a rigorously controlled hemispheric asymmetry paradigm to examine the effects of distractor faces presented outside the focus of attention (Experiment 2). No studies to date have presented distractor faces centrally behind target names to test the attention capture capacity of face identity on a naming task, and this experiment presents a novel approach to the problem of controlling the focus of attention.

### Aims Experiment 1

In Experiment 1, we test whether different types of face cause differential levels of distraction when processing one’s own name, a friend’s name and a stranger’s name, when presented inside the focus of attention. Of additional interest are the properties of a face which are responsible for capturing attention. Highly familiar faces may capture attention because they are “robustly represented” in the brain [1]. These robust representations are likely to rely heavily on configural information [28]. Recent evidence [3] suggests that self-faces may be processed in a qualitatively different way than other highly familiar faces, activating strong configural *and* featural processing. Specifically, while familiar face processing is detrimentally affected by inversion, which disrupts configural processing [29]–[31], processing speed advantages remain for self-faces when inverted. As such, the comparison of self-faces and other highly familiar faces in an attention capture task which includes upright and inverted faces should tell us much about the relative input of facial configural and featural information which are particularly implicated in attention capture.

If attention-capturing capacity is based largely on configural processing, then differences observed between upright friend and unfamiliar faces should disappear for inverted faces, because configural processing suffers with inversion. For self-faces, any attention-capturing capacity should remain for inverted faces, as we are particularly good at processing the self-face relative to other types of face when configural information is disrupted [3], [23]. Alternatively, if the attention-capturing capacity of familiar faces (self, friend) is based on another mechanism, inversion should have the same effect on friend and self-faces.

### Aims Experiment 2

In Experiment 2 we investigate how different types of face can attract attention when presented outside of the focus of attention (peripherally). There are a number of reasons why we are particularly interested in following up on previous reports of peripheral self-face attention capture. Firstly, differences in stimulus duration could account for discrepancies in accounts of peripheral self-face attention capture. Stimulus duration control is of importance here; a long stimulus duration potentially allows time for the participant to explicitly shift attention from the name to the face picture, which would elicit a shift in the locus of attention. In this experiment, we employ several measures to rigorously control focus of attention – peripheral stimuli are presented briefly enough to prevent an explicit shift of the focus of attention and a chin rest and participant eye-monitoring techniques are used to ensure that fixation does not shift towards the peripheral stimuli.

A second area of interest involves hemispheric presentation. A previous report of peripheral self-face attention capture found that the presentation location of the distractor face (to the left or right of the target stimulus) did not produce an effect [26]. Considering issues of hemispheric asymmetry in face processing in general (e.g., [32]) and self-face processing in particular (e.g., [16], [18]), this is surprising. In the current study, we manipulate hemispheric presentation in a tightly controlled manner. If the attention-grabbing capacity of faces is modulated by visual field presentation, we might expect them to produce more attentional interference when presented in the left visual field (right hemisphere; RH) relative to the right (left hemisphere; LH), as faces are processed preferentially in the RH (e.g., [33]). In addition, considering that self-face processing may activate a more bilateral neural network that other familiar faces [3], [15]–[20], LH interference may be increased for self-face relative to other face distractor trials.

## Experiment 1

### Method

#### Participants

Forty participants (24 female) with a mean age of 26.5 years (SD = 7.8) volunteered to take part in the study. Each participant was paired with a highly familiar same-sex friend whom they had known for at least one year, and whom they saw on a daily or almost daily basis. The majority of the participants were recruited in pairs, where each person served as a friend for the other participant. Data from six participants were discarded due to data coding errors (two participants) or participant error in understanding the instructions (four participants). The remaining 34 participants (21 female) had a mean age of 26.2 years (SD = 8.1).

#### Ethics Statement

Written informed consent was obtained from all participants prior to taking part in the study. The consent procedure and all other elements of both experiments detailed in this manuscript received full ethical approval from the Faculty Research Ethics Panel (Science and Technology) at Anglia Ruskin University. The approval number for both experiments is FST/FREP/11/17. The individuals pictured in the figures of this manuscript have given written informed consent (as outlined in PLOS consent form) to publish their images.

#### Stimuli

Participants were photographed in similar conditions under controlled lighting. Participants posed with a neutral expression while looking directly at the camera (Nikon D300). Using Adobe Photoshop, images were converted to greyscale and rotated to ensure that the eyes were collinear. An oval vignette (245×320 pixels) was applied to each facial image, ensuring that the jawline and hairline of each face were visible. Images were saved as normal and mirror-reversed copies. The mirror reversed copies of the images served as the “self” stimuli for participants, while the “friend” and “unfamiliar” stimuli were viewed normally (see 34 for evidence that these are the preferred views of self-faces and other familiar faces). Images were saved in both upright and inverted orientations.

**Figure 1:**
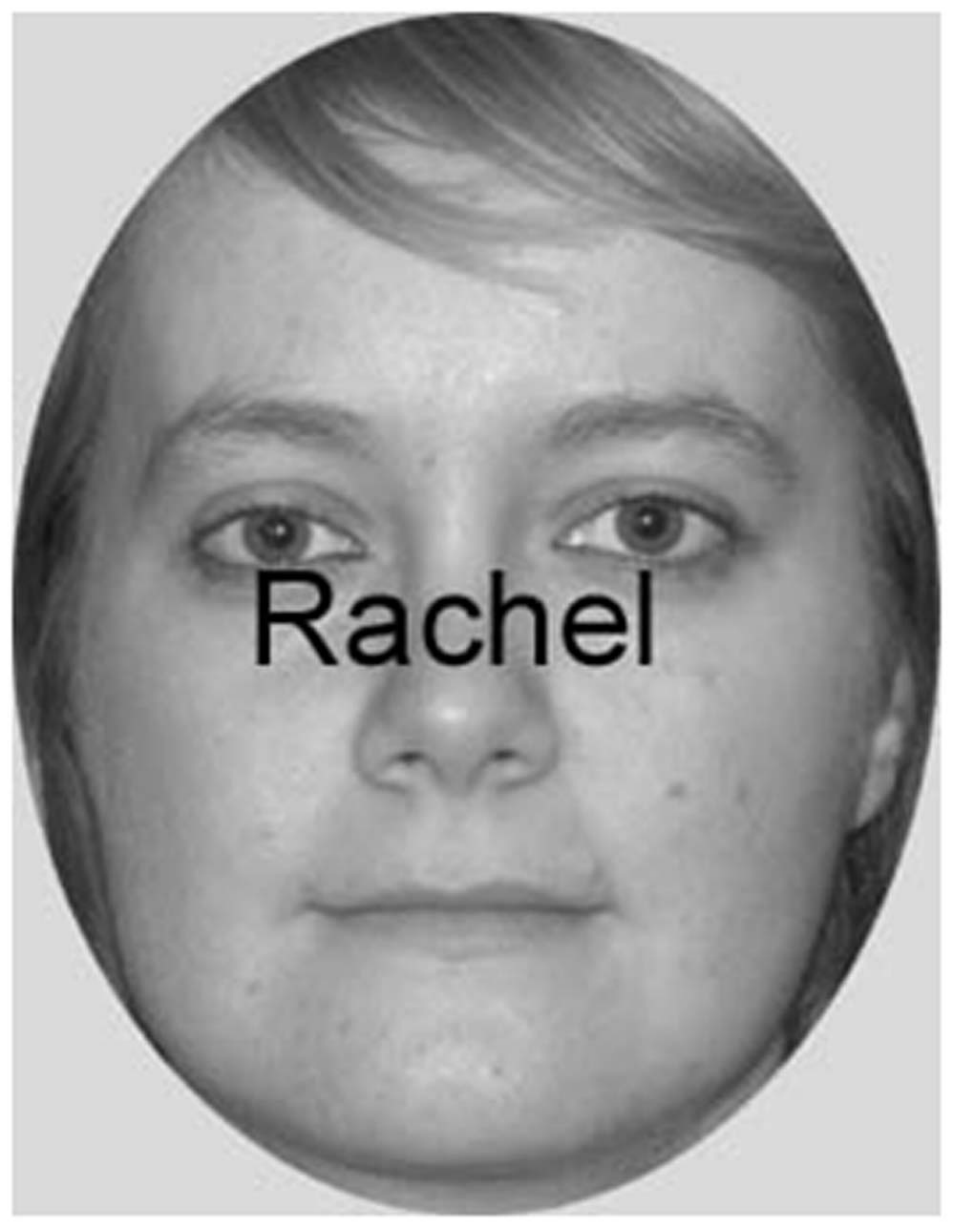
Example of an upright stimulus from Experiment 1.

Each participant’s set of stimuli comprised images of their own face, a friend’s face and a stranger’s face overlaid with a name. This name was their own name, their friend’s name or the stranger’s name. The name was placed centrally in an identical position across each facial image (centre of name at 160 pixels from bottom of image). Where an image of a face was presented in an inverted orientation, the text of the name was presented in upright orientation. Images were checked to ensure that the eyes and mouth were not obscured by text in any of the images (see Figure 1; the individual pictured here has given written informed consent for the use of this image). Images were viewed on a 17 inch screen of a Dell PC. Images subtended a viewing angle of 5.32 by 6.95 degrees when viewed from a distance of approximately 70 cm.

#### Procedure

Prior to testing, participants were shown upright versions of all three images (self [mirror-reversed], friend and stranger) without any text across the faces. The names identifying the faces were written on the screen below. Participants were asked to look at the faces for as long as it took for them to be confidently able to name each of the three faces. This was to ensure that participants were able to label the unfamiliar face with a name. This process took between 30–100 s for all participants.

Participants ran ten practice trials followed by a block of test trials. A trial comprised the presentation of a face (self, friend, unfamiliar) in either upright or inverted orientation with a name written across it (participant’s own name, friend’s name or stranger’s name). Participants were required to press a button on the keyboard (“c”, “v” or “b”) to indicate whether the name presented was their own name, their friend’s name or the stranger’s name. The order of the buttons allocated to “self”, “friend” and “stranger” was counterbalanced across participants. Stimuli were left on the screen until the participant responded. Each trial was followed by an inter-stimulus interval (ISI) varying between 500 and 1500 ms. Participants were instructed not to attend to the faces, and to respond as quickly and as accurately as possible.

Trials were balanced such that each face type (self, friend, stranger) was paired with each name type (self, friend, stranger) and equal number of times, and these pairings were presented with faces in upright and inverted orientations an equal number of times. Trials were presented in randomised order. The testing block comprised 216 trials (3 face types X 3 name types X 2 face orientations X 12 repetitions each).

### Results

Reaction times (RT) for correct responses were analysed. Incorrect responses accounted for 5.2% of the data, and were removed. For each participant, RT’s more than two standard deviations away from that participant’s mean were removed as outliers [35]; these accounted for 10.3% of trials. Data can be found at http://dx.doi.org/10.6084/m9.figshare.942382 [36].

A 3-way repeated-measures ANOVA was carried out, with factors of Distractor Face (self, friend, stranger), Target Name (self, friend, stranger) and Orientation (upright, inverted), and with RT to correct responses serving as the dependent variable. All post-hoc tests were interpreted using Bonferroni adjustment for multiple comparisons.

**Figure 2:**
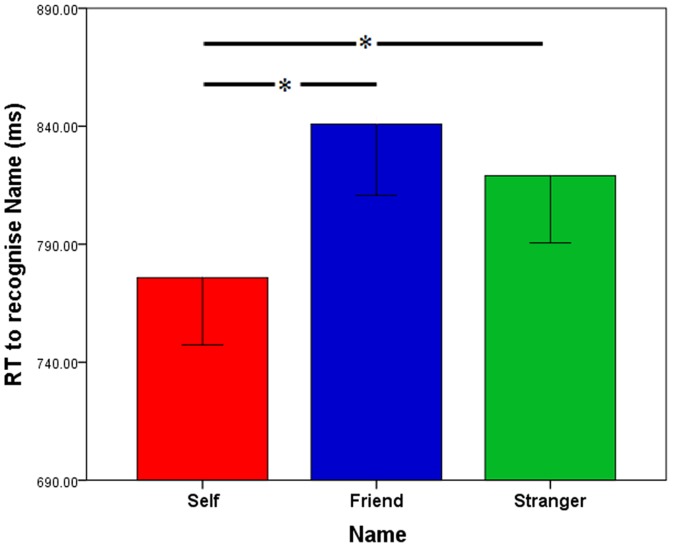
Response times to the self-name, friend’s name and stranger’s name in Experiment 1. Mean response times to recognise the self-name (red) a friend’s name (blue) and a stranger’s name (green) in Experiment 1.

Analysis revealed a significant effect of Target Name, *F*(2,66) = 10.44, *p*<.001, η_p_^2^ = .240, with a priori follow-up tests showing participants responding significantly faster to their own name than to a friend’s name, *t*(33) = 4.86, *p*<.05, *d* = .380, or a stranger’s name, *t*(33) = 2.56, *p*<.05, *d* = .260. RT in response to friend and stranger names did not differ, *t*(33) = 1.70, ns, *d* = .128 (see Figure 2).

There was no main effect of Distractor Face, *F*(2,66) = 0.69, ns., η_p_^2^ = .011, but this null effect is qualified by a significant interaction between Distractor Face and Target Name, *F*(4,132) = 6.84, *p*<.001, η_p_^2^ = .172. Follow-up tests show that when responding to a friend’s name, responses were significantly faster when the name was accompanied by the friend’s face (Target Name-Distractor Face congruence) relative to the self-face, *t*(33) = 2.69, *p*<.017, *d* = .171, or a stranger’s face, *t*(33) = 3.44, *p*<.017, *d* = .206 (Target Name-Distractor Face incongruence), suggesting that the presence of the friend’s face facilitated friend name processing. Responses to the friend’s name did not differ when accompanied by the self-face compared to the stranger’s face, *t*(33) = 0.55, ns, *d* = .042. Alpha is Bonferroni corrected to.017 for three comparisons.

Similarly, when responding to the self-name, responses were significantly faster when the name was accompanied by the self-face (Target Name-Distractor Face congruence) relative to a friend’s face, *t*(33) = 3.19, *p*<.017, *d* = .289, or a stranger’s face, *t*(33) = 2.40, *p* = .022, *d* = .217 (closely approaching significance at.017; Target Name-Distractor Face incongruence), suggesting that the presence of the self-face facilitated self-name processing. Responses to the self-name did not differ when accompanied by a friend’s face or a stranger’s face, *t*(33) = 0.94, ns, *d* = .063. Alpha is Bonferroni corrected to.017 for three comparisons.

**Figure 3:**
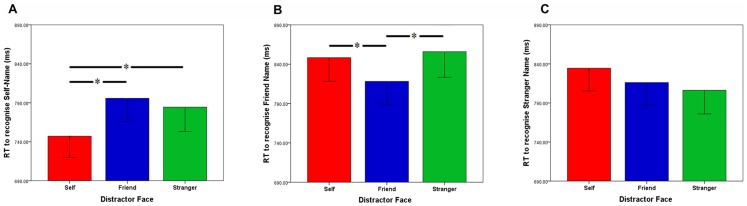
Influence of centrally presented task-irrelevant distractor faces on speed of name recognition. Mean response times to recognise the self-name (panel A) a friend’s name (panel B) and a stranger’s name (panel C) when the self-face (red), friend face (blue) and stranger face (green) was presented centrally as a distractor (Experiment 1).

When responding to the stranger’s name, no differences were observed depending on whether the Distractor Face presented was congruent or incongruent to the Target Name (self-face Vs stranger face, *t*(33) = 2.14, ns, *d* = .163; friend face Vs stranger face, *t*(33) = 0.70, ns, *d* = .058; self-face Vs friend face, *t*(33) = 1.61, ns, *d* = .108; alpha is Bonferroni corrected to.017 for three comparisons). Overall, congruent face-name stimuli pairings elicited faster naming responses than incongruent pairings for both Self and Friend pairings, but not for Stranger pairings. See Figure 3 for illustration of these interaction effects.

There was no effect of Orientation, *F*(1,33) = 2.92, ns, η_p_^2^ = .081,nor did Orientation interact with any of the other variables (Orientation by Target Name by Distractor Face: *F*(4,132) = 1.48, ns, η_p_^2^ = .043; Orientation by Target Name: *F*(2,66) = 1.82, ns, η_p_^2^ = .052; Orientation by Distractor Face: *F*(2,66) = 0.08, ns, η_p_^2^ = .003).

#### Accuracy Analyses

As predicted with a straightforward name-recognition task, there was a ceiling effect for accuracy, with participants correctly identifying whether a name was their own, a friend’s or a stranger’s at an accuracy rate of 97.56% (SD = 1.81). Here only one minor result reached significance, with participants being slightly less accurate when responding to a stranger’s name in the presence of a friend’s face (96.81%, SD = 4.82) compared to the stranger’s face (98.89%, SD = 2.13), *t*(33) = 3.25, *p*<.05. Considering the obvious ceiling effect in the accuracy data, we do not interpret this effect to be of importance, and do not discuss it further.

## Experiment 2

### Method

#### Participants

Thirty-nine participants (23 female) with a mean age of 25.5 years (SD = 6.4) volunteered to take part in the study. Again, each participant was paired with a highly familiar same-sex friend whom they had known for at least one year, and whom they saw on a daily or almost daily basis. Data from one participant were discarded due to data coding errors. The remaining 38 participants (23 female) had a mean age of 25.7 years (SD = 6.4). All participants were right-handed, as assessed by the Oldfield Inventory [37], with a mean laterality quotient of 87.6 (SD = 17.9). Left-handed and ambidextrous individuals were not invited to participate because hemispheric asymmetry in face processing was a variable of interest and the brains of right-handed individuals are considered to be more strongly and conventionally lateralised [38]. Full ethical approval was gained for this study, the details of which are outlined in the Methods section of Experiment 1.

#### Stimuli

**Figure 4:**
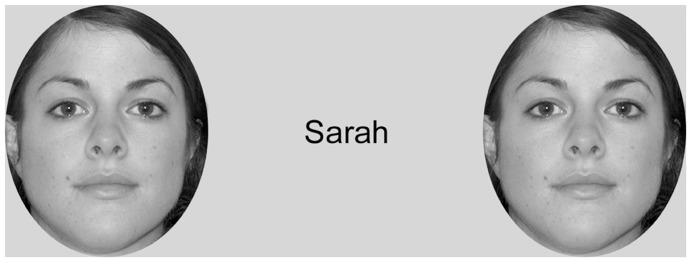
Example of a bilateral stimulus from Experiment 2.

Photographs of participants were collected and edited in a similar manner to Experiment 1. After conversion to greyscale and applying an oval vignette to each face image (370×460 pixels), a set of stimuli were created for each participant comprising images of their own face (mirror-reversed), a friend’s face and a stranger’s face. These faces were presented to the left (LVF), right (RVF) or both (bilaterally) of a centrally presented name. This name was their own name, their friend’s name or the stranger’s name, and was placed in an identical position (225 pixels from the bottom of the image) for each stimulus (see Figure 4; the individual pictured here has given written informed consent for the use of this image). Where images of faces were presented bilaterally, the faces were always identical. Images subtended a viewing angle of 7.76 by 9.80, and the centre of each image was 9.32 degrees to the left or right of the centre of the screen when viewed from a distance of 70 cm. This viewing distance was maintained by the use of a chinrest. To ensure that the face images were presented to each hemisphere (or both) in a controlled manner, the researcher monitored participants’ eye movements in real time using a webcam to check that their gaze remained on the centre of the screen at all times.

#### Procedure

Participants were initially familiarised with their three faces to be used as their stimuli along with their associated names in a similar manner to Experiment 1.

Participants ran ten practice trials followed by two blocks of test trials. One block of testing was completed with the right hand and the other with the left hand; the order of these blocks was counterbalanced across participants. A trial comprised the presentation of a name in the centre of the screen, with the participant’s own face, a friend’s face or a stranger’s face appearing to the left, to the right or on both sides of the name. The name presented was the participant’s own name, a friend’s name or a stranger’s name. The participant’s task was to indicate by pressing a button on the keyboard (“c”, “v” or “b”) who the name belonged to (self, friend, or stranger). The order of the buttons allocated to “self”, “friend” and “stranger” was counterbalanced across participants. Each stimulus was presented on screen for 250 ms, followed by an ISI varying between 500 and 1,500 ms. Participants were instructed not to attend to the faces, and to respond as quickly and as accurately as possible.

Trials were balanced such that each face type (self, friend, stranger) was paired with each name type (self, friend, stranger) and equal number of times, and these pairings were presented with faces in the LVF, the RVF and bilaterally an equal number of times. Trials were presented in randomised order. Each testing block comprised 189 trials (3 face types X 3 name types X 3 visual field presentations X 7 repetitions each).

### Results

Reaction times (RT) for correct responses were analysed. Incorrect responses accounted for 10.6% of the data, and were removed. For each participant, RT’s more than two standard deviations away from that participant’s mean were removed as outliers [35]; these accounted for 14.2% of trials. Data can be found at http://dx.doi.org/10.6084/m9.figshare.942383 [39].

A 3-way repeated-measures ANOVA was carried out, with factors of Distractor Face (self, friend, stranger), Target Name (self, friend, stranger) and Visual Field (LVF, RVF, bilateral), and with RT to correct responses serving as the dependent variable. All post-hoc tests were interpreted using Bonferroni adjustment for multiple comparisons.

**Figure 5:**
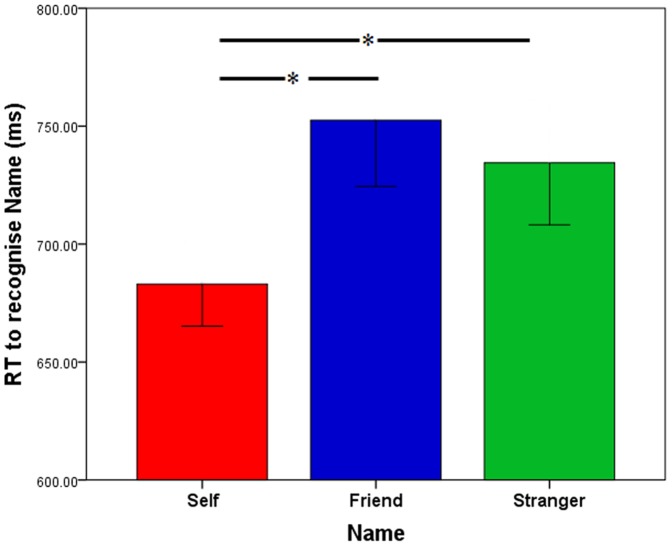
Response times to the self-name, friend’s name and stranger’s name in Experiment 2. Mean response times to recognise the self-name (red) a friend’s name (blue) and a stranger’s name (green) in Experiment 2.

Analysis revealed a significant effect of Target Name, *F*(2,74) = 13.12, *p*<.001, η_p_^2^ = .262, with a priori follow-up tests showing participants responding significantly faster to their own name than to a friend’s name, *t*(37) = 4.25, *p*<.05, *d* = .480, or a stranger’s name, *t*(37) = 3.29, *p*<.05, *d* = .372. RT in response to friend and stranger names did not differ, *t*(37) = 1.99, ns, *d* = .108. (see Figure 5).

**Figure 6:**
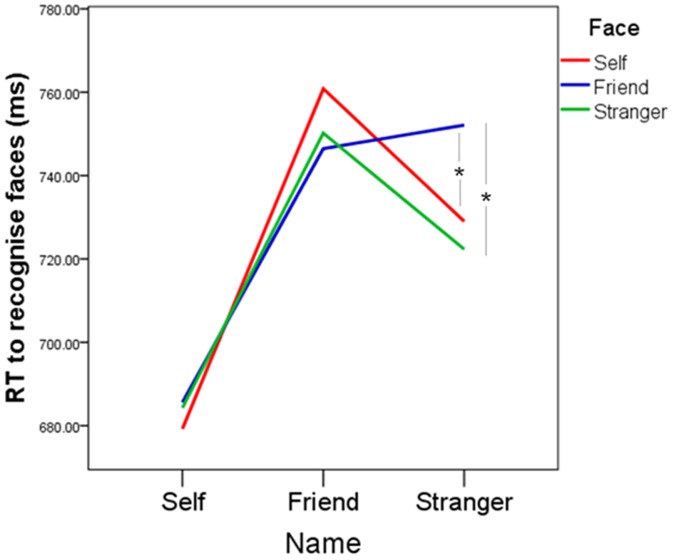
Influence of peripherally presented task-irrelevant distractor faces on speed of name recognition. Mean response times to recognise the self-name, a friend’s name and a stranger’s name in the peripherally presented presence of the self-face (red line), a friend’s face (blue line) and a stranger’s face (green line).

There was no main effect of Distractor Face, *F*(2,74) = 1.71, ns., η_p_^2^ = .044, but this null effect is qualified by a significant interaction between Distractor Face and Target Name, *F*(4,148) = 3.95, *p*<.005, η_p_^2^ = .096. Follow-up tests show that type of Distractor Face did not have any effect when responding to the self-name or a friend’s name, but when responding to a stranger’s name the presence of a friend’s face significantly increased RT relative to both the stranger’s face, *t*(37) = 3.12, *p*<.017, *d* = .175, and the self-face, *t*(37) = 2.71, *p*<.017, *d* = .135, suggesting that a peripherally presented friend’s face causes more distraction when processing a stranger’s name than either the self-face or a stranger’s face. There was no difference in effect when responding to a stranger’s name in the presence of the self-face or stranger’s face, *t*(37) = 1.06, ns, *d* = .044. Alpha is Bonferroni corrected to.017 for three comparisons. See Figure 6 for illustration of the interaction effects.

There was no effect of Visual Field, *F*(2,74) = 0.67, ns., η_p_^2^ = .018, nor did Visual Field interact with any of the other variables (Visual Field by Target Name by Distractor Face: *F*(8,296) = 0.63, ns., η_p_^2^ = .017; Visual Field by Target Name: *F*(4,148) = 0.29, ns., η_p_^2^ = .008; Visual Field by Distractor Face: *F*(4,148) = 1.09, ns., η_p_^2^ = .029).

#### Accuracy Analyses

As expected, there was a ceiling effect for accuracy, with participants correctly identifying whether a name was their own, a friend’s or a stranger’s at an accuracy rate of 97.18% (SD = 1.82). There were no significant effects for accuracy in Experiment 2.

### Cross-Experimental Analyses between Experiments 1 and 2

Examining the data suggested that responses to names were faster in Experiment 2 (when distractor faces were presented peripherally) compared to Experiment 1 (when distractor faces were presented centrally). In order to examine whether the robust self-name RT advantage observed in both experiments differed depending on whether distractor faces were presented centrally or peripherally, additional cross-experimental analyses were carried out, with factors of Experiment (1, 2), Target Name (self, friend, stranger) and Distractor Face (self, friend, stranger).

There was a significant effect of Experiment, with participants responding significantly faster to all types of name in Experiment 2 (distractor presented outside the focus of attention; 723.34 ms, SE = 24.64) compared with Experiment 1 (distractor presented inside the focus of attention; 811.91, SE = 26.05), *F*(1, 70) = 6.10, *p*<.05, η_p_^2^ = .080. This suggests a greater interference effect of all types of distractor faces on all types of name when distractors were presented centrally rather than peripherally.

There was no significant interaction effect between Target Name and Experiment or Distractor Face and Experiment, suggesting similar self-name RT advantages across both experiments, regardless of where distractor faces were presented. A significant Target Name by Distractor Face by Experiment interaction mirrored what findings from Experiments 1 and 2 showed separately – for Experiment 1, congruent face-name stimuli pairings elicited faster naming responses than incongruent pairings for both Self and Friend pairings, but not for Stranger pairings and for Experiment 2, congruence did not facilitate naming but rather a friend’s face provided more distraction than either the self-face or a stranger’s face when responding to a stranger’s name.

## Discussion

Across two studies, we found that participants responded significantly faster to their own name than to other names. The self-face did not cause more distraction than other faces either when presented centrally or peripherally, suggesting that our own face does not selectively grab our attention when either inside or outside the focus of attention. Instead, we report a facilitation effect of familiar faces on congruent familiar name recognition when those faces are inside the focus of attention, and an interesting distraction effect when friend faces are presented outside the focus of attention.

### Self-name processing

A strong finding across both studies was that participants responded significantly faster to their own name than to a friend’s or stranger’s name. This finding opposes Brédart and Devue’s [26] report that the self-name and a classmate’s name were identified equally quickly. One possible reason for this discrepancy is that our task was simpler – in the current experiments the participants simply had to identify the name presented, whereas in Brédart and Devue’s study [26] the name to be identified was presented in tandem with a letter string. Indeed, when others have used a simple identity decision task, they also report that the self-name elicits faster responses than other familiar names [40]. We interpret our finding as a straightforward self-referential effect, with speeded processing for the self-name due to its importance as a self-referential stimulus. In this way, our finding mirrors previous reports of speeded self-face processing relative to other faces (e.g., [1]–[3]).

### Our own face does not grab our attention

Importantly, the self-face did not cause any more distraction than either a friend’s face or a stranger’s face, either when presented inside (Experiment 1) or outside (Experiment 2) the focus of attention. We conclude from this that our own face does not automatically or selectively capture our attention. Devue and Brédart [14] argue that the self-face is a better example of a self-referential stimulus than the self-name, as the self-name can be shared with others, whereas the self-face is truly unique to the self. However, the self-name is often used to capture our attention in a real-world setting (i.e. someone may call you by your name to attract your attention), making us particularly sensitive to its presence. The self-face – though an important self-referential stimulus – is not normally used to capture our attention in this way. The self-face may be processed as “special” in several ways – as demonstrated by speeded processing [1]–[3] and a more bilateral neural representation compared to other faces [15]–[20] – but it does not appear to have special attention capturing properties. The speeded processing afforded to self-referential stimuli and observed here and elsewhere does not appear to be driven by automatic attention capture.

The findings outlined in this paper are important to theories of self-referential processing because they suggest that not all self-referential stimuli are equal. While it is established that the self-name automatically and selectively captures attention [9]–[12], our findings show that this is not the case for the self-face. Different types of self-referential stimuli (name, face) may well serve different purposes, particularly in terms of capturing attention. It is both important and interesting to contrast ways in which various types of self-referential stimuli interact with attentional mechanisms and processing speeds. Our findings suggest that in the field of self-referential processing, more focus should be placed on *types* of self-referential stimuli and examining the different purposes preferential processing of these stimuli could serve.

### Inside the focus of attention

When presented inside the focus of attention (Experiment 1), task-irrelevant self-face stimuli did not cause more distraction than other types of face. Our findings somewhat contradict Gronau and colleagues [12] in this respect, who showed that centrally presented task-irrelevant self-referential stimuli (the self-name) caused more distraction than other names. In our study, the centrally presented self-faces (and friend’s faces) had a very different effect on naming speeds – these highly familiar faces facilitated processing of their associated names. That is, responses to the self-name were faster in the presence of the self-face and responses to a friend’s name were faster in the presence of the friend’s face. This suggests that the task-irrelevant faces were being processed; they just did not capture attention in a selective way. Indeed, rather than cause distraction, under certain conditions congruent faces facilitated name recognition. Importantly, both the self-face and a friend’s face facilitated the processing of their associated names when presented inside the focus of attention, while this was not true for unfamiliar faces. This suggests that the facilitation effect of face presentation on name recognition may occur for all highly familiar faces, and is not self-specific. Tong and Nakayama [1] propose that we develop particularly efficient processing skills for highly familiar, robustly represented faces, and we consider the facilitated processing for congruent familiar face-name pairs reported here to be evidence of this.

### Outside the focus of attention

When faces were presented outside the focus of attention (Experiment 2), an interesting phenomenon emerged. The self-face did not selectively grab attention, as previously reported [26]. Instead, a friend’s face selectively captured attention – as demonstrated by significantly increased reaction time to recognise a stranger’s name in the presence of a friend’s face. We interpret this as a “social importance” effect. In a real-world setting, it would be sensible to be primed to pick out familiar friends’ faces outside the focus of attention – for example, in a crowd. This would not be the case for our own face, or a stranger’s face. That a friend’s face only caused distraction when processing a stranger’s name (and not the self-name or a friend’s name) supports this interpretation. Both the self-name and a friend’s name are socially interesting stimuli to us, and so we invest our attention in them. However, a stranger’s name is not an interesting social stimulus, and so our attention can more easily be captured by a socially relevant stimulus – a friend’s face.

With several studies – including our own – now demonstrating that the self-face is not more attention-grabbing than other types of face, observing a friend face attention-grabbing effect at peripheral presentation is not wholly surprising. However, cautious interpretation is warranted here as this effect was not predicted in our initial hypotheses. Our interpretation of a “social importance” effect is tentative and warrants a further programme of study, perhaps varying the degree of social importance of the distractor face.

### Facilitation versus distraction effects

We report facilitation effects for familiar faces in Experiment 1, when distractor faces were presented inside the focus of attention, but a selective distraction effect for friend faces in Experiment 2, with distractor faces presented outside the focus of attention. We posit that this difference is based on cognitive capacity, which might vary based on the focus of attention.

When distractor faces are presented centrally behind target names, it is likely that there is sufficient capacity to process the distractor faces as well as responding to the target name. Indeed, all types of face (self, friend, stranger) presented inside the focus of attention automatically and non-selectively grabbed attention. This is evidenced by significantly slower response times in the name identification task in in Experiment 1 compared to Experiment 2, where faces were presented outside the focus of attention, and suggests that the presence of faces in general interferes with name identification when those faces are presented inside the focus of attention. That all types of face (self, friend, stranger) capture attention when presented centrally makes the observed differential facilitation effects possible. If we have sufficient cognitive capacity to attend to distractor faces presented inside the focus of attention, the simultaneous presentation of two congruent identity cues – face and name – should lead to speeded processing of the target stimulus. Indeed, this is what was observed in Experiment 1, for robustly represented familiar faces.

Conversely, when distractor faces are presented outside the focus of attention, cognitive capacity during the name identification task may not stretch to easily processing the faces while responding to the target names. In this case, it seems that not all faces have the capacity to grab attention – only a friend’s face captured attention, and then only when responding to a socially unimportant stimulus (a stranger’s name).

### Orientation result

Surprisingly, the orientation of a task-irrelevant face did not have any effect on its ability to facilitate name recognition for robustly represented faces (Experiment 1). This may be because robustly represented faces (self and friend) should contain some view-invariant information [1], allowing them to convey information in both upright and inverted orientations. We were expecting inversion to affect self-face processing to a lesser degree than other familiar face processing [3], [23], as self-face processing may be less dependent on configural processing than other familiar face processing [3], [21]–[23]. However, while inversion may affect self-face and other familiar face processing differentially in tasks where the face is attended and task-relevant, it appears that a robustly represented task-irrelevant face’s ability to facilitate naming is not affected by inversion. This suggests that whatever information is driving the facilitation effect is not tied to configural processing.

### Hemispheric presentation

Similarly, we observed no main effect of hemispheric presentation on the ability of faces to capture attention (Experiment 2). This finding is surprising, considering the dominance of the RH in processing faces (e.g., [33]), but it does support a previous report that the visual field presentation of faces did not affect attention capture [26]. Considering the known hemispheric effects involved in face processing in general and self-face processing in particular, we conclude that task-irrelevant faces presented outside of the focus of attention do not recruit the same processing resources as the task relevant face usually used in studies of hemispheric asymmetry. In this instance, processing the task-irrelevant peripherally presented faces may have been too secondary to the central attentional task for normal hemispheric advantages to be observed. Additionally, the necessity to present the distractor images peripherally led us to choose a large angular distance of 9.32 degrees. While traditional hemispheric effects for face processing can be observed at this angular distance [3], it exceeds the angular distance used in many hemispheric asymmetry studies, and may have lessened any effects of hemispheric presentation. Further study varying angular distance would be useful in informing as to when faces in general can be more fully processed (providing a facilitation effect) versus when faces can selectively provide a distraction.

### Conclusion

We conclude that speed of processing advantages commonly observed for self-faces [1]–[3] are not driven by automatic attention capture. In two experiments, we demonstrate no distracting effect of the self-face in a name recognition task. Instead, we demonstrate a facilitation effect whereby robustly represented faces (self, friend) speed the processing of familiar names (self and friend, respectively; Experiment 1). This is not true for unfamiliar faces, which do not have robust neural representation. Thus it appears that face-name facilitation is only possible after a robust facial representation has developed. When faces are presented outside of the focus of attention, facilitation no longer occurs. Instead, we observe a significant attention grabbing effect of familiar friend faces when processing strangers’ names (Experiment 2). We interpret this as a “social importance” effect, whereby we may be tuned to pick out and pay attention to familiar friend faces in a crowd. Finally, across both experiments the self-name was processed faster than other names, indicating the importance of this self-referential stimulus. It is unlikely that speed of processing advantages for self-face stimuli are tied to their attention-grabbing properties. We propose that any “special” status the self-face holds in the brain may instead be ascribed to a functional uniqueness in terms of how the self-face is processed once attended.
